# A new monoclonal antibody that blocks dimerisation and inhibits *c-kit* mutation-driven tumour growth

**DOI:** 10.1007/s00432-020-03490-6

**Published:** 2021-01-03

**Authors:** Chenguang Bai, Yi Xu, Cen Qiu

**Affiliations:** 1 Department of Pathology, Ninth People’s Hospital, Shanghai Jiao Tong University School of Medicine, Number 639 Zhizaoju Rd., Shanghai, 200001 China; 2 Department of Pathology, Changhai Hospital, Second Military Medical University, Shanghai, China; 3 Department of Pathology, Yueyang Integrative Medicine Hospital, Shanghai University of Traditional Chinese Medicine, Shanghai, China

**Keywords:** Gastrointestinal stromal tumour, Monoclonal antibody, Imatinib-resistant, Dimerisation

## Abstract

**Purpose:**

Imatinib, a small-molecule tyrosine kinase inhibitor, has shown good clinical activity by inhibiting adenosine triphosphate (ATP) binding to the receptor. Unfortunately, majority of patients eventually develop drug resistance, which limits the long-term benefits of the tyrosine kinase inhibitors and poses a significant challenge in the clinical management of GIST. The aim of our study was to explore the feasibility of blocking KIT dimerisation upstream of the phosphorylation in imatinib-resistant GIST.

**Method:**

KITMAb was prepared using hybridoma technique. The biological function of KITMAb was examined in KIT-dimer-expressing cells constructed by transfecting with liposomes using enzyme linked immunosorbent assay (ELISA), immunohistochemistry, western blot, MTT, Annexin V/FITC, and flow cytometry assay, respectively.

**Results:**

KIT-dimer was expressed in 293 cells transfected with *c-kit* mutated-type pcDNA3.1. Treatment of KIT-dimer-expressing cells with the KITMAb significantly decreased the expression of both KIT-dimer and other phosphorylated proteins of KIT downstream signalling pathway. Furthermore, KITMAb slowed down cell growth and reduced the proportion of cells in the proliferative phase (S + G2-M). Finally, we also found that KITMAb treatment accelerated cell apoptosis. These results indicate that KITMAb strongly inhibits KIT receptor dimerisation-mediated signalling pathway and cell growth responses in vitro*.*

**Conclusions:**

We demonstrate *c-kit* mutation-driven KIT auto-dimerisation prior to tyrosine kinase phosphorylation as same as the procedure in ligand-dependent signalling pathway and describe a monoclonal antibody, KITMAb, with strong affinity to the dimerisation domain of KIT that blocks the important step in both the KIT signalling pathways. Further, the results suggest that treatment with KITMAb may be potentially therapeutic in imatinib-resistant GIST.

**Supplementary Information:**

The online version contains supplementary material available at 10.1007/s00432-020-03490-6.

## Introduction

Gastrointestinal stromal tumour (GIST) is the most common mesenchymal tumour of the digestive tract. Most GISTs are caused by gain-of-function mutations in *c-kit* that trigger intrinsic receptor tyrosine kinase activity and downstream signalling cascades including phosphatidylinositol 3-kinase (PI3K)/protein kinase B (AKT) pathways even in the absence of the binding of its ligand, stem cell factor (SCF) (Hirota et al. 1998; Chiao et al. 2019; Lennartsson et al. 1999; Lev et al. 1992; Serve et al. 1994). Although the small-molecule tyrosine kinase inhibitor, imatinib, has shown optimum clinical activity in *c-kit*-driven GIST patients, the effects are often limited because of intrinsic and acquired resistance resulting in tumour recurrence and progression in most patients (Schroeder et al. 2020). To overcome primary and secondary resistance to imatinib, more effective KIT-targeted therapies are urgently needed.

KIT belongs to the transmembrane growth factor receptor family with an activation mechanism that involves the crosslinking of two receptors following SCF binding (Besmer et al. 1986; Yarden et al. 1987). Under normal conditions, in the ligand-dependent receptor signalling pathway, the intrinsic tyrosine kinase phosphorylation is followed by receptor dimerisation. However, we detected the expression of KIT-dimer in 293 cells transfected with pcDNA3.1 expressing mutated-type *c-kit*. These results demonstrated that the *c-kit* mutation drove auto-dimerisation, and promoted receptor phosphorylation, and ligand-independent receptor signalling pathway. Therefore, dimerisation is the common step in both the activation processes of KIT prior to phosphorylation and therefore, blocking receptor dimerisation may be more effective than blocking the phosphorylated receptor.

Based on the design of pertuzumab, a monoclonal antibody that sterically blocks Her-2-dimerisation, we prepared a murine monoclonal antibody, KITMAb, which specifically binds to the dimerisation domain of KIT (the fourth and the fifth extracellular motifs) (Yuzawa et al. 2007). The results showed that KITMAb inhibits the expression of KIT-dimer and cell growth responses due to receptor dimerisation. Taken together, our findings confirmed that KITMAb inhibits dimerisation upstream of the phosphorylation in KIT signalling pathway suggesting the potential of dimerisation blocking therapy in imatinib-resistant GIST patients.

## Materials and methods

### Cell lines, human tissues and animals

Human embryonic kidney cells (HEK 293 cells) were obtained from the Laboratory of Thoracic Surgery, Changhai Hospital, Shanghai (China). Cells were cultured in Dulbecco’s modified eagle medium (DMEM) with 10% foetal calf serum (GIBCO BRL, Grand Island, NY, USA) at 37.5 °C in a humidified 5% CO_2_ atmosphere. The four plasmid vectors used in our study including blank pcDNA3.1, *c-kit* wild-type pcDNA3.1, *c-kit* mutated-type pcDNA3.1, and pcDNA6.2 were stored in our laboratory. A total of 5 GIST samples obtained from the department of pathology of Ninth People's Hospital affiliated to Jiao Tong University School of Medicine, Shanghai (China) underwent examination to identify KITMAb and the diagnoses were confirmed by two pathologists (Bai CG and Qiu C) (The clinical characteristics were summarised in Table 1S). Imatinib was purchased from Novartis Pharma, Basel, Switzerland and the mouse IgG antibody was purchased from Amyjet Scientific, China. Female BALB/c mice (8 weeks-old) were obtained from the Experimental Animal Centre of the Second Military Medical University.

### Design, preparation, and purification of KITMAb

The antigen primers for KIT extracellular domains 4 and 5 (that are involved in receptor dimerisation) were synthesised using polymerase chain reaction (PCR) according to the protocol described in previous studies (Yuzawa et al. 2007) based on its sequence from the GenBank. The cDNA fragment of interest obtained from the recombination plasmid of *c-kit* wild-type pcDNA3.1 was ligated to vector PET28 and then transformed into DH5a competent cells. The positive bacterial clones were selected using streptomycin and electroporated into *Escherichia coli* BL21 cells. Expression of the antigenic protein was induced using isopropyl-β-D-thiogalactopyranoside (IPTG), collected by centrifugation, and purified using nickel affinity chromatography. KITMAb was prepared by immunising BALB/c mice with the mixture of the antigenic proteins using hybridoma technique (Reuben 1982). Ascetic fluid from the mice was analysed for antibody secretion using enzyme linked immunosorbent assay (ELISA). KITMAb was purified using protein G affinity chromatography.

### Generation of cells expressing the KIT-dimer

PcDNA3.1 containing the *c-kit* gene was transfected into 293 cells using Lipofectamine 2000 reagent (Invitrogen, Carlsbad, CA, USA). To observe the transfection efficiency directly under a fluorescence microscope, the cells were cotransfected with pcDNA6.2 harbouring green fluorescent protein. Stably transfected cell lines were isolated through limiting dilution in the presence of 400 μg/mL G418.

### Mutational analysis of the *c-kit* gene

Genomic DNA was extracted from cells using a genomic DNA isolation kit (BioVison, Milpitas, CA, USA). The sequences of the primers used for PCR were as follows: forward, 5′-CCAGAGTGCTCTAATGACTG-3′ and reverse, 5′-AGCCCCTGTTTCATACTGAC-3′. The PCR conditions were performed in a PCR system (Thermo Fisher Scientific, Waltham, MA, USA). The PCR products were directly subjected to sequence analysis.

### Western blot analysis

Frozen GIST samples were calibrated and homogenised in lysis buffer (20 mM Tris, 150 mM NaCl, 1 mM orthovanadate, 10 mM NaF, 1 mM phenylmethanesulphonyl fluoride (PMSF), 0.5 μg/mL leupeptin, 1 μg/mL pepstatin, 10 KIU/mL aprotinin, 1% triton X-100). Lysates were rocked at 4 °C for 30 min and then centrifuged at 12,000 rpm for 15 min. The supernatant protein concentration was measured using a BCA protein assay kit (Abcam, Cambridge, MA, USA).

Cells were plated at a density of 2 × 10^5^ cells/well in 6-well plates and serum starved overnight. The following day, the cells were either treated in the presence or absence of IgG, KITMAb or imatinib at varying doses for 72 h at 37 °C. The cells were rinsed in phosphate-buffered saline (PBS) and lysed in 1 × cell lysis buffer.

The expression of KIT-dimer was detected by western blotting using native-PAGE; whereas, the expression of KIT-monomer was detected by western blotting using SDS-PAGE. The blots were incubated with primary antibody dilutions: 1:2000 KIT antibody (DAKO, A4502, Glostrup, Denmark), 1:1000 phospho-KIT, 1:1000 phospho-MAPK, 1:1000 phospho-AKT (Cell Signaling Technology, 3391, 9102, 9275, Danvers, MA, USA), and 1:3000 glyceraldehyde-3-phosphate dehydrogenase (GAPDH, Abcam, 6C5, Cambridge, MA, USA).

### Immunohistochemical analysis

Immunohistochemical staining of anti-KIT (1:500, DAKO, A4502, Glostrup, Denmark) was performed using EnVision™ system (DAKO, Glostrup, Denmark) according to the manufacturer's instruction.

### Analysis of cell cycle and apoptosis

A total of 1 × 10^6^ mutated-type cells were collected, treated with 75% alcohol, washed with PBS, and resuspended in 10 μL propidium iodide solution. The cells were dissociated with RNaseA (Thermo Fisher Scientific, Waltham, MA, USA), stained with Annexin V/FITC, and then analysed by flow cytometry.

### Statistical analysis

Data analysis was performed using SPSS26.0 (IBM, Armonk, NY, USA). *P* < 0.05 was considered to be statistically significant (*α* = 0.05).

## Results

### Identification of the gene that encodes the dimerisation region of KIT

**Fig. 1:**
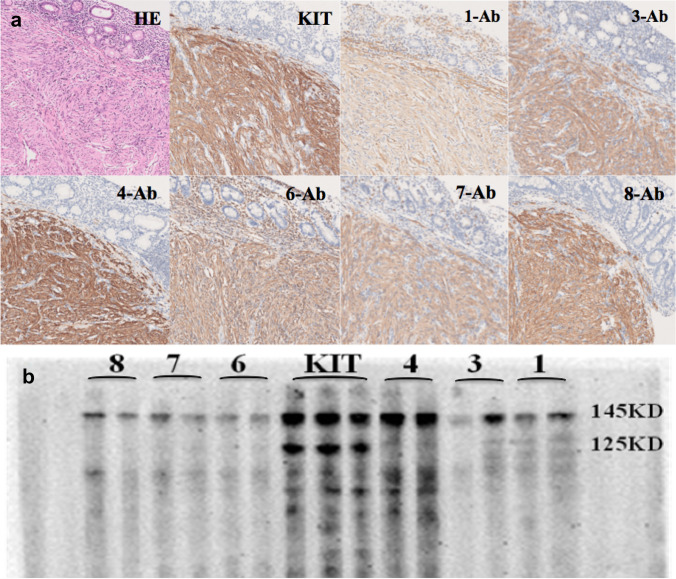
Identification of KITMAb. **a** Identification of KITMAb by immunohistochemical analysis (Case 1). **b** Identification of KITMAb by SDS-PAGE (Case 1)

KIT is characterised by the presence of an extracellular region with five immunoglobulin (Ig)-like motifs, of which the fourth and fifth motifs are involved in dimerisation (Lev et al. 1992; Serve et al. 1994; Yuzawa et al. 2007). Therefore, based on the *c-kit* gene sequence from the GenBank, primers were designed to span this region as follows: antigen 1–1 expressed proteins in the fourth and fifth extracellular motifs, antigen 1–2 expressed proteins in the fourth extracellular motif and antigen 1–3 expressed proteins in the fifth extracellular motif. The upstream primers were 5′-CGCGGATCCCCCATGATAAACACTACAGT-3′, 5′-CGCGGATCCCCCATGATAAACACTACAGT-3′ and 5′-CGCGGATCCCCAGAAATCCTGACTTACGA-3′. All the restriction enzyme cutting sites were BamHI. The downstream primers were 5′-CCGCTCGAGTGCAAAGTTAAAATAGGCAG-3′, 5′-CCGCTCGAGATTCACATAAACATTAAATG-3′ and 5′-CCGCTCGAGCAGGGTGTGGGGATGGATT-3′. All the restriction enzyme cutting sites were XhoI. After amplification of the corresponding cDNA fragments of three antigens from *c-kit* wild-type pcDNA3.1 using PCR, the gene segments were analysed using restriction mapping and localised between 100 and 250 bp, consistent with the expected sizes, in agarose gel electrophoresis (Fig. 1S). Analysis of sequence homology using BLAST software revealed that the sequence of the amplified *c-kit* gene was identical to that published in the GenBank.

### Induction of expression and purification of the recombinant antigen

**Fig. 2:**
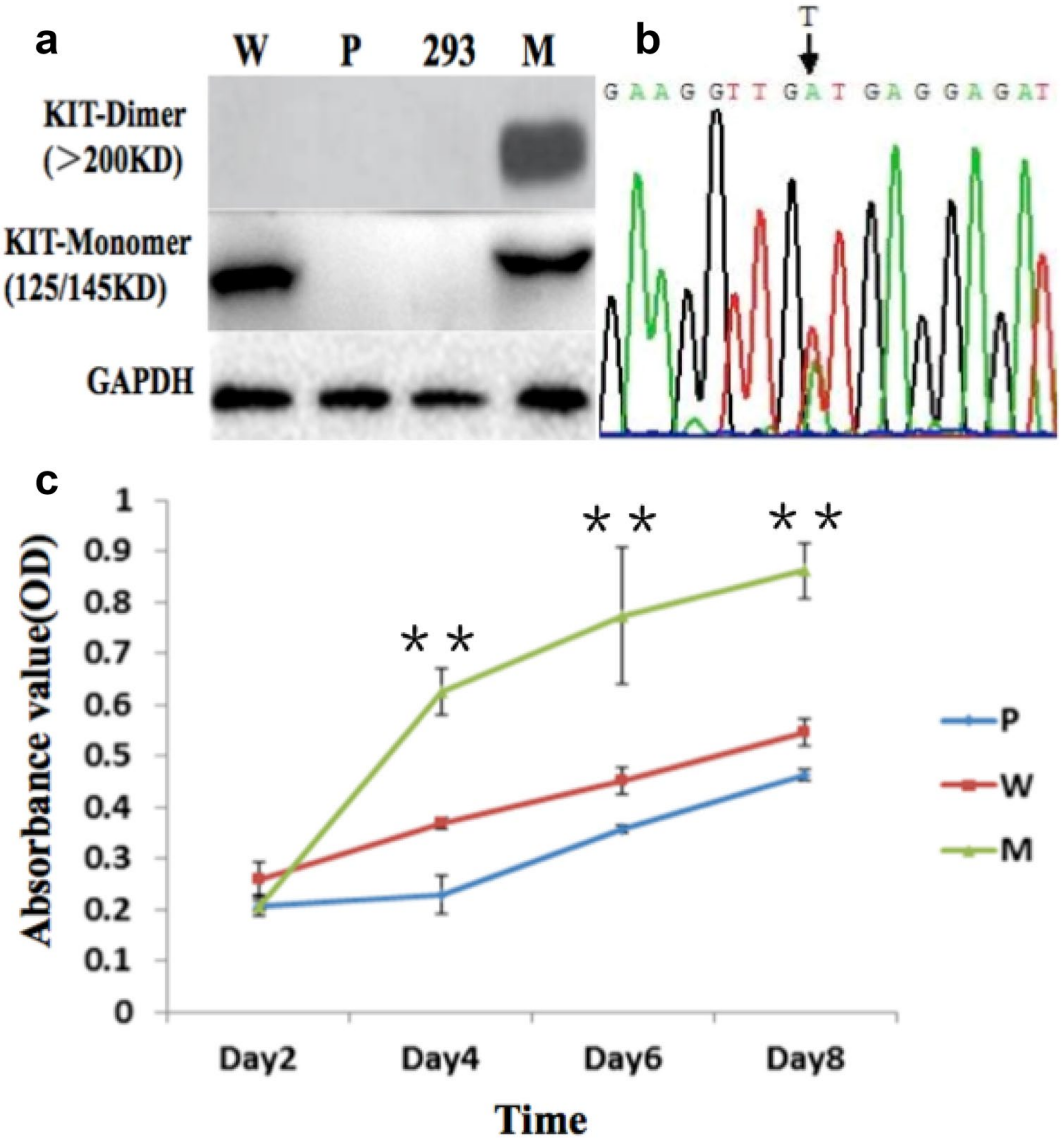
Establishment of KIT-dimer-expressing cell lines. **a** The expression of KIT-dimer in untransfected and transfected 293 cells detected by western blotting using native-PAGE whereas the expression of KIT-monomer detected by western blotting using SDS-PAGE (M meant 293 cells transfected with *c-kit* mutated-type pcDNA3.1, W meant 293 cells transfected with *c-kit* wild-type pcDNA3.1, P meant 293 cells transfected with blank pcDNA3.1, and 293 meant untransfected 293 cells). **b** DNA sequence analysis of exon 11 mutation (point mutation in V560D, GTT → GAT) in *c-kit* mutated-type pcDNA3.1 transfected 293 cells. **c** Cell proliferation of untransfected and transfected 293 cells detected using MTT (M meant 293 cells transfected with *c-kit* mutated-type pcDNA3.1, W meant 293 cells transfected with *c-kit* wild-type pcDNA3.1, and P meant 293 cells transfected with blank pcDNA3.1). *P < 0.05, **P < 0.01

Sequence information of antigens generated was as follows: the sequence of antigen 1–1 that localised to 317–507 aa was PMINTTVFVNDGENVDLIVEYEAFPKPEHQQWIYMNRTFTDKWEDYPKSENESNIRYVSELHLTRLKGTEGGTYTFLVSNSDVNAAIAFNVYVNTKPEILTYDRLVNGMLQCVAAGFPEPTIDWYFCPGTEQRCSASVLPVDVQTLNSSGPPFGKLVVQSSIDSSAFKHNGTVECKAYNDVGKTSAYFNFA (molecular weight: 21.44 kDa). The sequence of antigen 1–2 that localised to 317–401 aa was PMINTTVFVNDGENVDLIVEYEAFPKPEHQQWIYMNRTFTDKWEDYPKSENESNIRYVSELHLTRLKGTEGGTYTFLVSNSDVNAAIAFNVYVN (molecular weight: 10.93 kDa). The sequence of antigen 1–3 that localised to 413–517 aa was PEILTYDRLVNGMLQCVAAGFPEPTIDWYFCPGTEQRCSASVLPVDVQTLNSSGPPFGKLVVQSSIDSSAFKHNGTVECKAYNDVGKTSAYFNFAFKEQIHPHTL (molecular weight: 11.53 kDa). The products of the antigenic proteins were mainly in the form of inclusion bodies following IPTG induction of the proteins. The molecular weights of three purified antigenic proteins were 26.01, 16.13, and 16.55 kDa, respectively, by western blotting with SDS-PAGE (Fig. 2S). These results were consistent with the molecular size of the designed antigenic protein. The concentration of three antigenic proteins analysed using BCA protein assay was 0.5, 0.3, and 0.4 mg/mL, respectively, and the purity of the antigens was 85, 80, and 90%, respectively.

### Identification of KITMAb

After successful generation of a set of 11 murine monoclonal antibodies named KITMAbs that block the KIT receptor dimerisation domain, the effective titres of the KITMAbs were analysed using ELISA (Table 1). The titre marked in bold was an effective titre (determination criterion: dilution value of OD450 greater than two times of the negative control and greater than 0.25). The results of the immunohistochemical assay indicated that six of the eleven KITMAbs (1-Ab, 3-Ab, 4-Ab, 6-Ab, 7-Ab, and 8-Ab) bound to KIT proteins in all the GIST tissues optimally, and the positive colour rendering was located in the cytoplasm or membrane of the GIST cells (Fig. 1a). Furthermore, KIT protein was extracted from GIST tissues, lysed, and KITMAb was identified by western blotting following SDS-PAGE. The results indicated that the selected KITMAbs bound to KIT protein extracted from the 5 GIST samples very well, and the positive bands were of the molecular weight of 145/125 kDa (Fig. 1b).

**Table 1:** Titer of KITMAb

Number	3.125 K	6.25 K	12.5 K	25 K	50 K	100 K	200 K	400 K	800 K	PC	NC
1	2.638	2.448	2.088	1.36	0.776	0.446	**0.252**	0.162	0.118	2.82	0.07
2	2.699	2.688	2.677	2.61	2.390	2.044	1.334	0.890	**0.507**	2.84	0.07
3	2.439	2.388	2.272	1.99	1.496	0.933	0.560	**0.364**	0.225	2.58	0.09
4	2.541	2.524	2.456	2.42	2.200	1.890	1.505	0.976	**0.625**	2.69	0.08
5	2.663	2.687	2.671	2.50	2.464	2.007	1.443	0.821	**0.487**	3.08	0.05
6	2.674	2.517	2.517	2.22	1.856	1.215	0.669	**0.345**	0.195	2.88	0.06
7	2.624	1.968	1.191	**0.53**	0.291	0.191	0.124	0.098	0.080	3.08	0.18
8	1.300	0.750	0.426	**0.29**	0.167	0.163	0.129	0.090	0.077	2.24	0.1
9	2.643	2.688	2.590	2.46	1.893	1.401	0.903	0.548	**0.360**	2.97	0.1
10	2.688	2.565	2.430	2.09	1.490	0.978	0.564	**0.328**	0.224	2.96	0.09
11	2.093	1.937	1.814	1.69	1.338	1.229	0.973	0.639	**0.372**	1.62	0.09

*PC* Positive Control, *NC* Negative Control

### Establishment of KIT-dimer-expressing cell lines

**Fig. 3:**
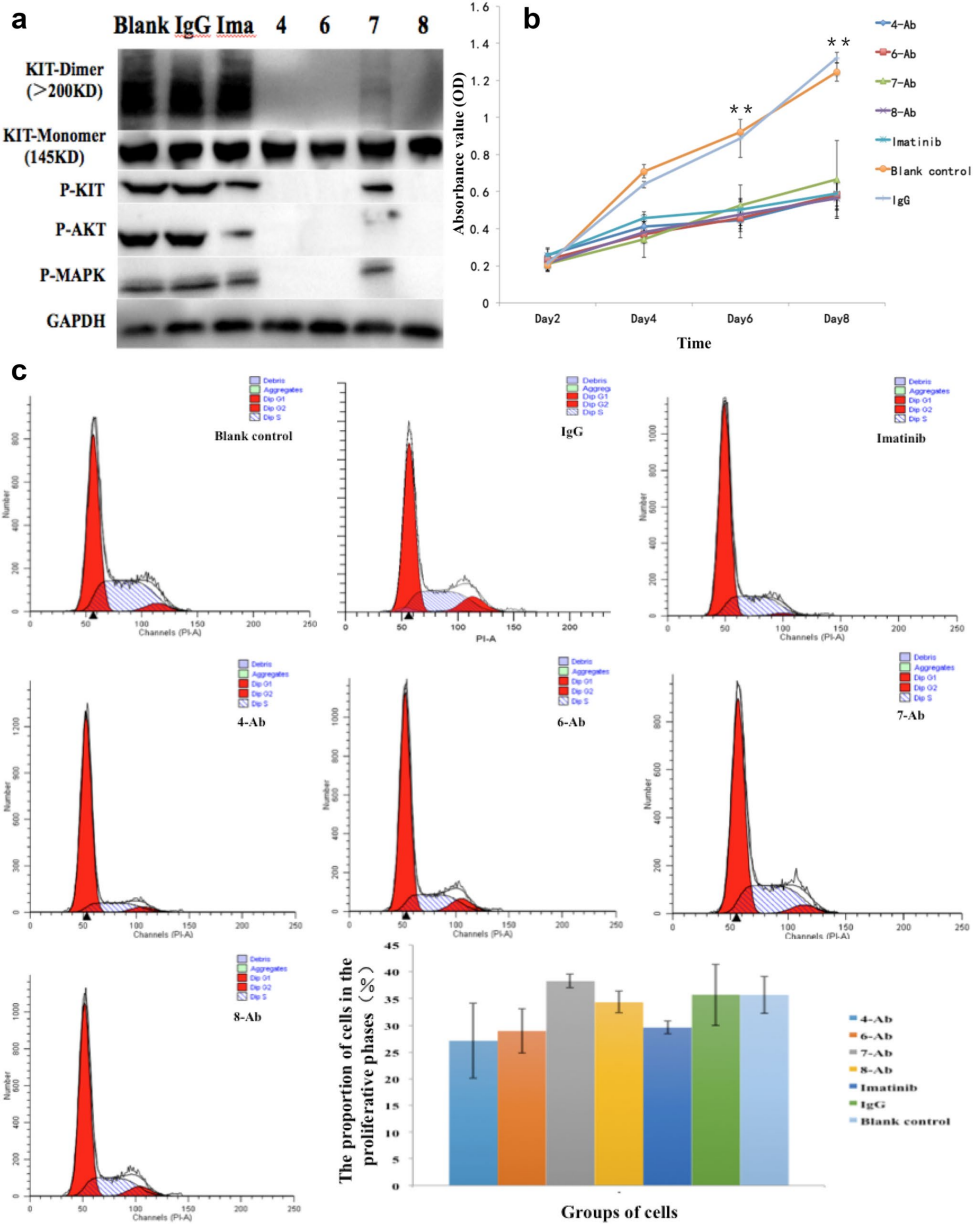

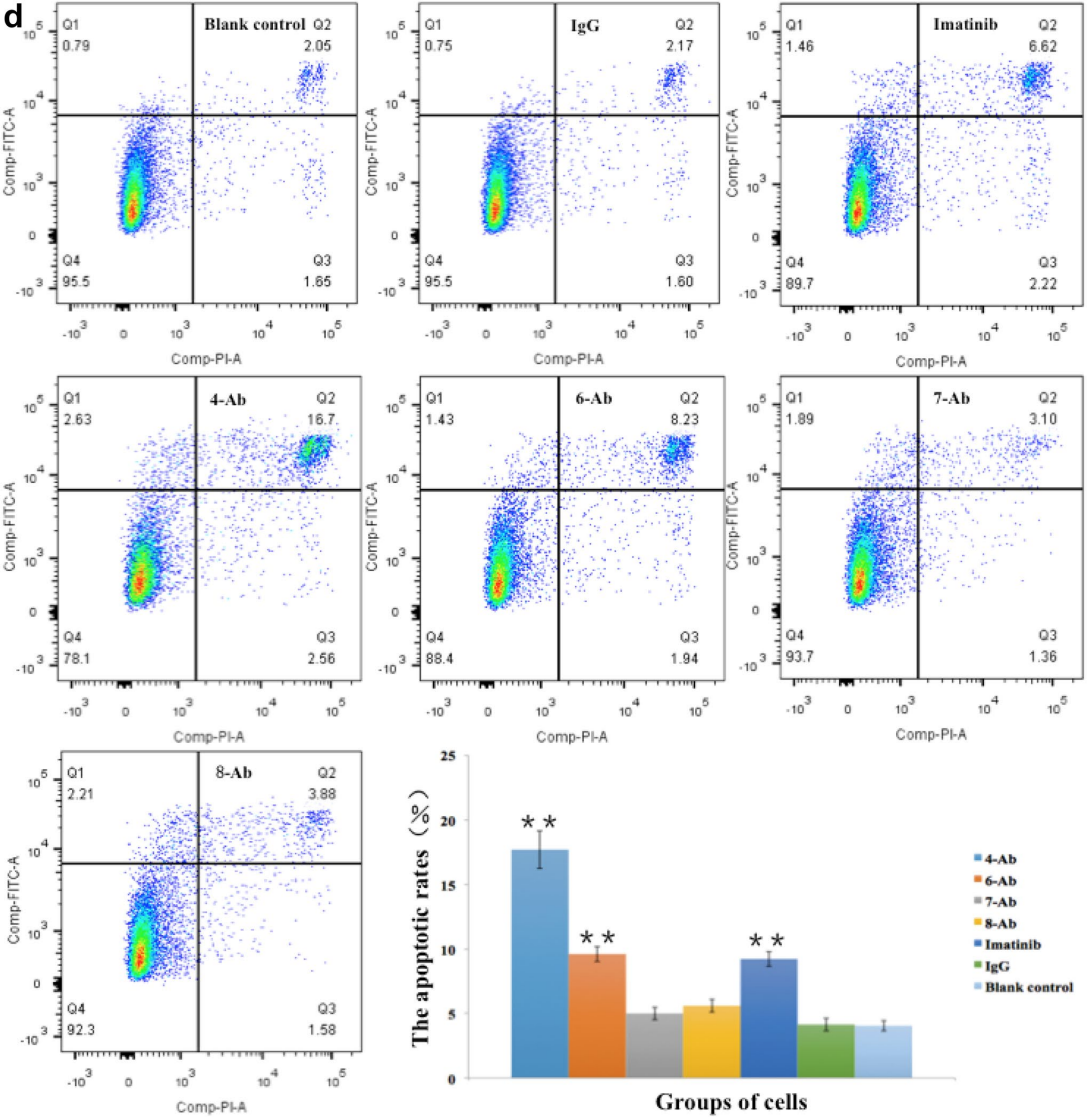
Detection of inhibition of KITMAb in KIT-dimer-expressing cell lines in vitro. **a** Detection of KIT-dimer in 293 cells transfected with *c-kit* mutated-type pcDNA3.1 by native-PAGE and detection of KIT-monomer by SDS-PAGE. **b** Cell proliferation of untreated, IgG-treated, imatinib-treated, and KITMAb-treated KIT-dimer-expressing cells detected using MTT assay. **c** Cell cycle in untreated, IgG-treated, imatinib-treated, and KITMAb-treated KIT-dimer-expressing cells detected using flow cytometry. **d** Cell apoptosis in untreated, IgG-treated, imatinib-treated, and KITMAb-treated KIT-dimer-expressing cells detected using Annexin V staining. *P < 0.05, **P < 0.01

Fluorescence microscopy revealed that the transfection efficiency was approximately 50% 48 h following transfection (Fig. 3S). The expression of KIT-monomer and -dimer was investigated by western blotting using SDS-PAGE and native-PAGE respectively. As shown in Fig. 2a, both mature KIT-monomer (145 kDa) and KIT-dimer (> 250 kDa) were expressed in 293 cells transfected with *c-kit* mutated-type pcDNA3.1; while, only unmature KIT-monomer (125 kDa) was expressed in 293 cells transfected *c-kit* wild-type pcDNA3.1. No KIT expression was detected in blank pcDNA3.1-transfected or untransfected 293 cells. MTT colorimetric assay was also conducted to measure cell proliferation in each cell line. There was no significant difference in the OD at 490 nm between 293 cells transfected with *c-kit* wild-type pcDNA3.1 and blank pcDNA3.1. Interestingly, the OD value of 293 cells transfected with *c-kit* mutated-type pcDNA3.1 was much higher that of the other two groups of 293 cells (*P* < 0.01, *F* = 101.593, Fig. 2c). These results confirmed that mutated *c-kit* gene promotes cell proliferation. DNA sequence analysis revealed no *c-kit* mutation in the 293 cells transfected with *c-kit* wild-type pcDNA3.1; while, exon 11 point mutation was detected in 293 cells transfected with *c-kit* mutated-type pcDNA3.1 (Fig. 2b).

### Inhibition of receptor dimerisation in KIT-expressing cell lines in vitro

**Fig. 4:**
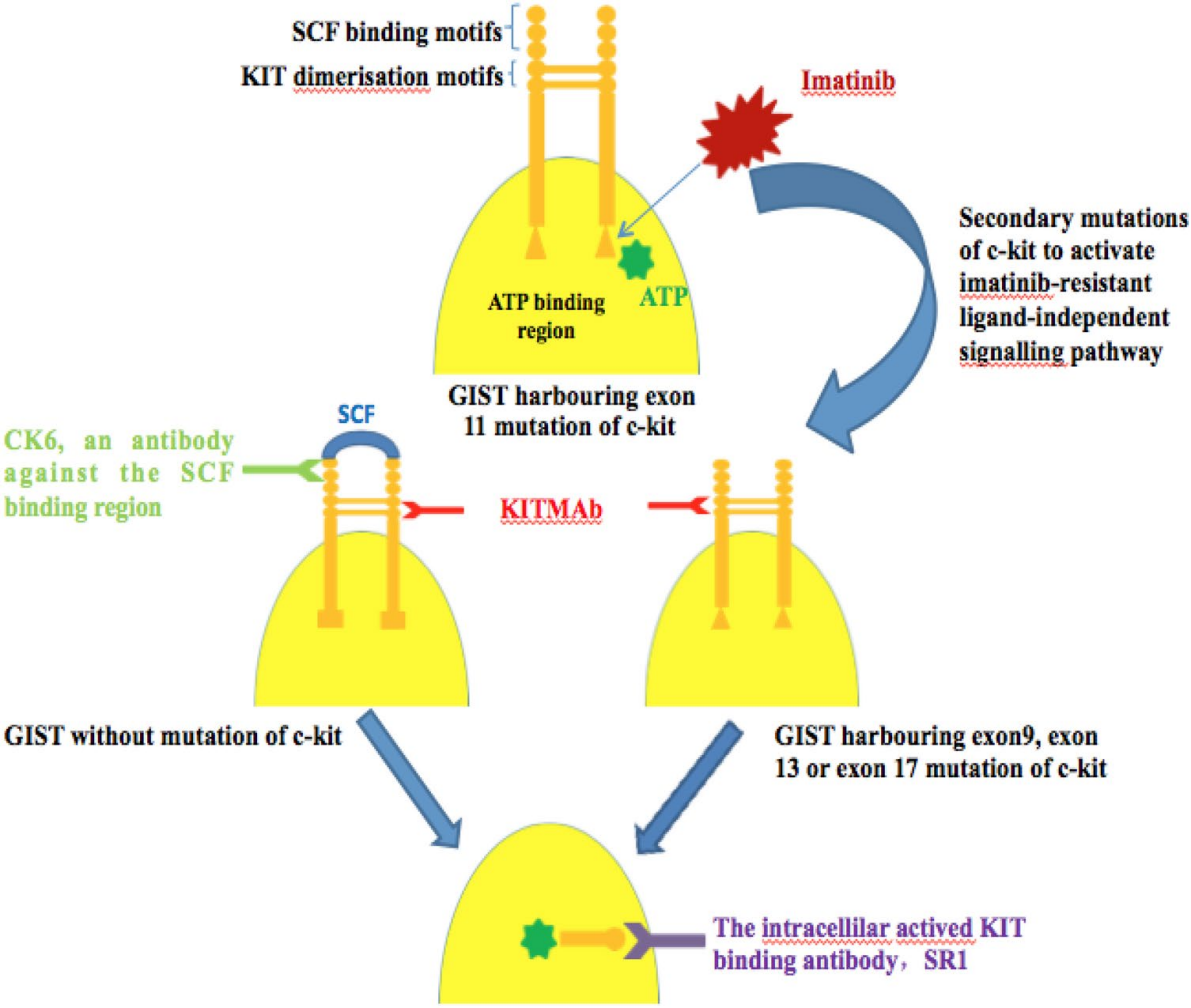
Use of KITMAb to bypass imatinib resistance in gastrointestinal stromal tumours

Following dimerisation of the receptor, the KIT protein becomes phosphorylated and the downstream signalling pathway is activated. Therefore, to measure the activation of the receptor, detecting the expression of KIT-dimer is more informative than detecting the expression of phosphorylated KIT because KIT has several phosphorylation sites. KITMAb was used at the concentration of 0.5 μg/mL (based on the clinically effective therapeutic dose of imatinib and the results of drug effective concentration screening test by using MTT assay depicted in Fig. 4S) in our study. Four of the previously selected KITMAbs (4-Ab, 6-Ab, 7-Ab, and 8-Ab) were found to significantly decrease the expression of KIT-dimer in 293 cells transfected with *c-kit* mutated-type pcDNA3.1 after 72 h of treatment as detected by western blot analysis of the corresponding cell lysates using native-PAGE. Pretreatment with KITMAbs also inhibited dimerisation stimulation of KIT signal transduction with reduced phospho-KIT, phospho-MAPK and phospho-AKT protein levels but not control mouse IgG and blank control. To demonstrate the specificity of the antibodies, imatinib was used as a positive control and it showed a bright band of KIT-dimer protein expression. In addition, there was no significant difference in the expression level of KIT-monomer protein in the different cell samples (Fig. 3a). Our finding suggests that KITMAb strongly binds to the dimerisation domain of KIT and results in inhibition of KIT receptor dimerisation. On the other hand, imatinib was found to have little effect on inhibition of KIT dimerisation.

To examine the ability of KITMAb to inhibit cell growth induced by dimerisation of KIT in vitro, cell proliferation, cell cycle, and apoptotic assays were performed. 293 cells expressing KIT-dimer were exposed for 72 h to either KITMAb or imatinib as experimental group or positive control, respectively. Untreated 293 cells expressing KIT-dimer were used as a blank control and cells treated with the mouse IgG were used as a negative control. Cell proliferation activity was measured using MTT assay and the results are depicted in Fig. 3b. Cell proliferation activity in all the four experimental groups and positive control group of cells was significantly lower than that of the blank control group of cells (*P* < 0.01, *F* = 72.193) and the negative control group of cells (*P* < 0.01, *F* = 90.554). Further observation revealed that the cell growth in the experimental groups decreased more compared to that of the positive control group, although the differences were not statistically significant (*P* = 0.065, *F* = 1.131). Neither KITMAb nor imatinib had any effect on cell proliferation of *c-kit* wild-type pcDNA3.1-transfected and untransfected 293 cells (data not provided here).

The results of cell cycle assay showed that the proportion of cells in the proliferative phases (S + G2-M) in blank control untreated KIT-dimer-expressing 293 cells, negative control cells treated with IgG, positive control cells treated with imatinib, and four experimental groups of cells exposed to KITMAb were 36.68 ± 8.31%, 35.72 ± 5.73%, 29.59 ± 1.21%, 27.13 ± 7.02%, 28.96 ± 4.11%, 38.26 ± 1.27%, and 34.32 ± 2.05%, respectively (Fig. 3c). Compared to the blank and the negative control group, the proportion of proliferative cells in the positive control and the four experimental groups decreased although the difference was not statistically significant when tested using ANOVA (*P* = 0.099, *F* = 2.252).

The results of the apoptotic assay showed that the apoptotic rates of the blank control, the negative control, the positive control, and four experimental groups were 4.07 ± 0.40%, 4.15 ± 0.50%, 9.26 ± 0.58%, 17.70 ± 1.45%, 9.64 ± 0.59%, 5.02 ± 0.49%, and 5.62 ± 0.50% (Fig. 3d). Compared to the blank and the negative control group, apoptosis was higher in the positive control and the four experimental groups (*P* < 0.01, *F* = 135.506). Further analysis using least significant difference T test (LSD-t) showed that apoptosis rates of experimental groups treated with 4-Ab, 6-Ab, 8-Ab and positive control group were significantly higher than that of the blank and the negative control group (Table 2). Compared to the positive control group treated with imatinib, apoptosis was higher in the experimental group treated with 4-Ab (*P* < 0.01). Apoptosis in group treated with 6-Ab was not statistically different from that in the positive control group (*P* = 0.539); whereas, apoptosis rates of experimental groups treated with 7-Ab and 8-Ab were lower than that of the positive control group (*P* < 0.01).

**Table 2:** The *P* value of LSD-t analysis

	4-Ab vs	6-Ab vs	7-Ab vs	8-Ab vs	Ima vs
Blank control	< 0.01	< 0.01	0.132	0.021	< 0.01
Negative control	< 0.01	< 0.01	0.168	0.027	< 0.01

## Discussion

Aberrant activation of the ligand-independent KIT signalling axis has been implicated in mutated-type GIST and accounts for 80% of all GIST cases (Hirota et al. 1998; Chiao et al. 2019). Small-molecule tyrosine kinase inhibitors resemble adenosine triphosphate (ATP) structurally and competitively bind to the ATP-binding domain of KIT, thus contributing to good clinical efficacy in GIST patients harbouring constitutively phosphorylated KIT receptors (Apsel et al. 2019). Unfortunately, the effects are limited based on the mutation site of *c-kit*. Only patients harbouring exon 11 mutation of *c-kit* are sensitive to imatinib therapy; while, other ligand-independent KIT signalling pathways activated by mutations in exon 9, exon 13, exon 17, and others are not blocked by imatinib. Further, primary drug resistance mainly appears in cases without mutation of *c-kit* (Florou et al. 2019; Ding et al. 2020; Hung et al. 2019). In addition, long-term imatinib application induces secondary mutations to activate new ligand-independent KIT signalling pathways that are insensitive to imatinib and lead to secondary resistance eventually (Raut et al. 2018; George and Chandrajit 2019). A functional antibody that targets receptor dimerisation is a viable alternative approach to treat imatinib-resistant GISTs, as it would inhibit both ligand-dependent and ligand-independent receptor signalling pathways.

In this study, we applied this strategy of blocking receptor dimerisation toward treatment of GIST and prepared a monoclonal antibody, KITMAb, that targets the receptor dimerisation domain. The properties of the antigen designed for KITMAb were consistent with the result in the GenBank and this guaranteed specific binding of KITMAb to KIT. To characterise the binding properties of KITMAb further, we used liposome-mediated transfection to first obtain KIT-dimer-expressing cell lines. The results demonstrated that KIT auto-dimerisation owing to the *c-kit* mutation rendered the 293 cells more proliferative, similar to the observation in GISTs. The expression of KIT-dimer was significantly decreased after treatment with KITMAb in vitro, which confirmed that KITMAb precisely blocked the dimerisation of KIT receptor. As a consequence of decreased expression of KIT-dimer, treatment with KITMAb reduced the protein levels of phospho-KIT, phospho-MAPK and phospho-AKT in KIT signal transduction. Besides, KITMAb reduced cell proliferation and cell cycle progression, and promoted cell apoptosis of KIT-dimer-expressing cells. Consistent with our expectation, KITMAb blocked the receptor dimerisation-activated KIT signalling pathway.

The development of monoclonal antibody-based therapy effectively addresses the significant challenge associated with imatinib-resistant GIST. However, the activated KIT binding antibody, SR1, lacks extensive in vivo characterisation and has agonistic activity owing to its large molecular weight (Edris et al. 2013). Another fully human IgG1 monoclonal antibody, CK6, blocks the interaction of KIT with SCF, but only interferes with ligand-stimulated signalling and cell growth responses (Maria et al. 2014). Unlike SR1 that antagonises the intracellular activated receptor, KITMAb binds to the extracellular region (Fig. 4). This implies that KITMAb does not need to enter the tumour cells to reach the effective concentration, which can be affected by molecular weight of the antibody. Furthermore, blocking of both the ligand-dependent and -independent signalling pathways by KITMAb is likely to exert greater clinical effect than blocking of only the ligand-dependent signal pathway as with CK6 (Fig. 4).

In summary, we describe the anti-tumour growth properties of KITMAb, a monoclonal antibody with affinity for the dimerisation region of KIT. KITMAb represents a potential therapeutic agent that can be effectively used to treat imatinib-resistant GIST patients.

## Conclusion

KIT dimerisation is prior to tyrosine kinase phosphorylation and drives GIST cancer regardless of *c-kit* mutation. Unlike imatinib whose effects are limited based on the mutation site of *c-kit*, KITMAb can inhibit dimerisation upstream of the phosphorylation in both ligand-dependent and -independent KIT signalling pathway. Our findings support the continued evaluation of KITMAb as potential therapy in imatinib-resistant GIST patients.

## Supplementary Information

Below is the link to the electronic supplementary material.

Supplementary file1 (DOCX 38 KB)

Supplementary file2 (DOCX 1440 KB)

## Data Availability

All data generated or analysed during this study are included in this published article.
