# Supplementary material for: A new monoclonal antibody that blocks dimerisation and inhibits c-kit mutation-driven tumour growth
Source: J Cancer Res Clin Oncol. 2021 Jan 3;147(4):1065–75. doi: 10.1007/s00432-020-03490-6 (PMC7954730; doi:10.1007/s00432-020-03490-6)
Supplement: Supplementary file 1 — Supplementary file1 (DOCX 38 KB) [file 432_2020_3490_MOESM1_ESM.docx]

**Table 1S Clinical characteristics of GIST samples**

| **Case Number** | **Age** | **Gender** | **Localization of tumour** |
| --- | --- | --- | --- |
| 1 | 37 | Male | Stomach |
| 2 | 52 | Male | Stomach |
| 3 | 49 | Male | Stomach |
| 4 | 66 | Female | Stomach |
| 5 | 62 | Female | Stomach |
