# Supplementary material for: A new monoclonal antibody that blocks dimerisation and inhibits c-kit mutation-driven tumour growth
Source: J Cancer Res Clin Oncol. 2021 Jan 3;147(4):1065–75. doi: 10.1007/s00432-020-03490-6 (PMC7954730; doi:10.1007/s00432-020-03490-6)
Supplement: Supplementary file 2 — Supplementary file2 (DOCX 1440 KB) [file 432_2020_3490_MOESM2_ESM.docx]

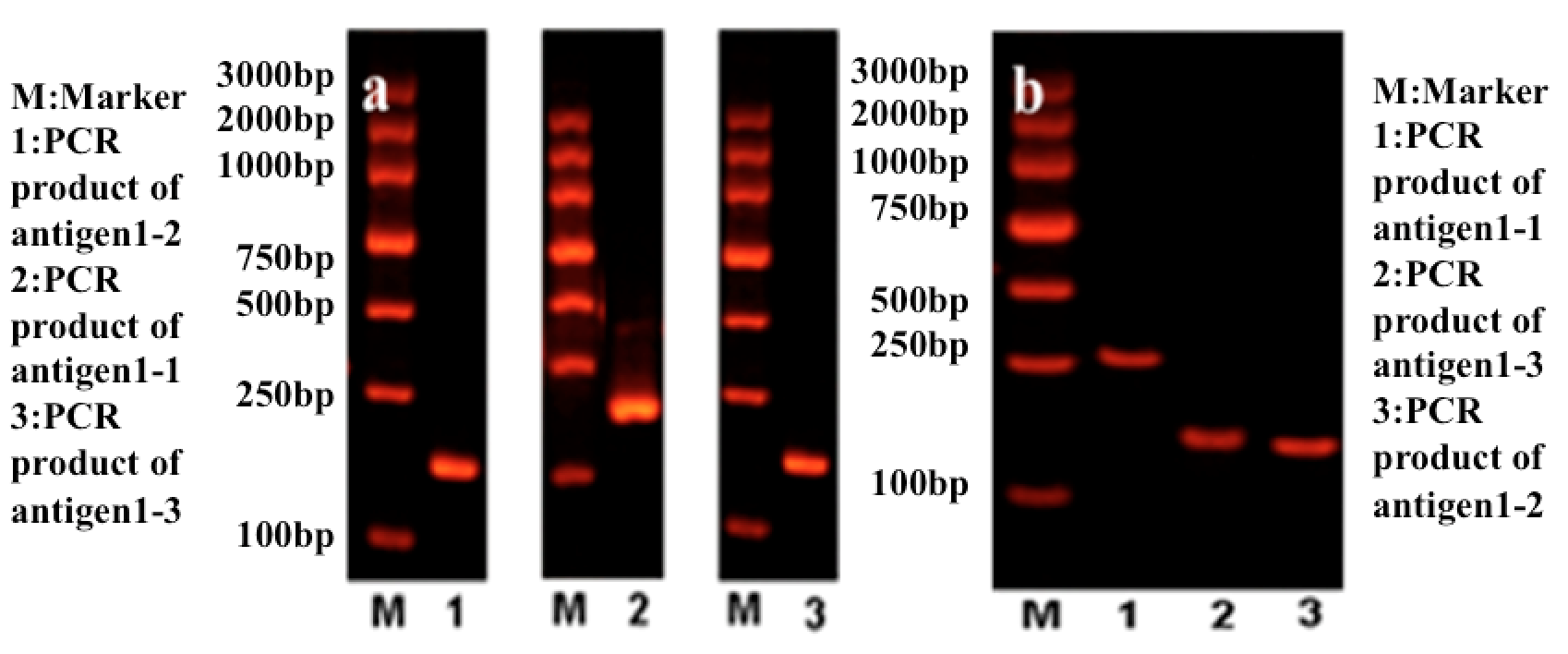


**Fig. 1 Restriction map of recombinant plasmids containing antigen-related cDNA fragments. (a) PCR products from positive bacterial colonies were identified by agarose gel electrophoresis. (b) The enzymatic digestion product was identified by agarose gel electrophoresis.**


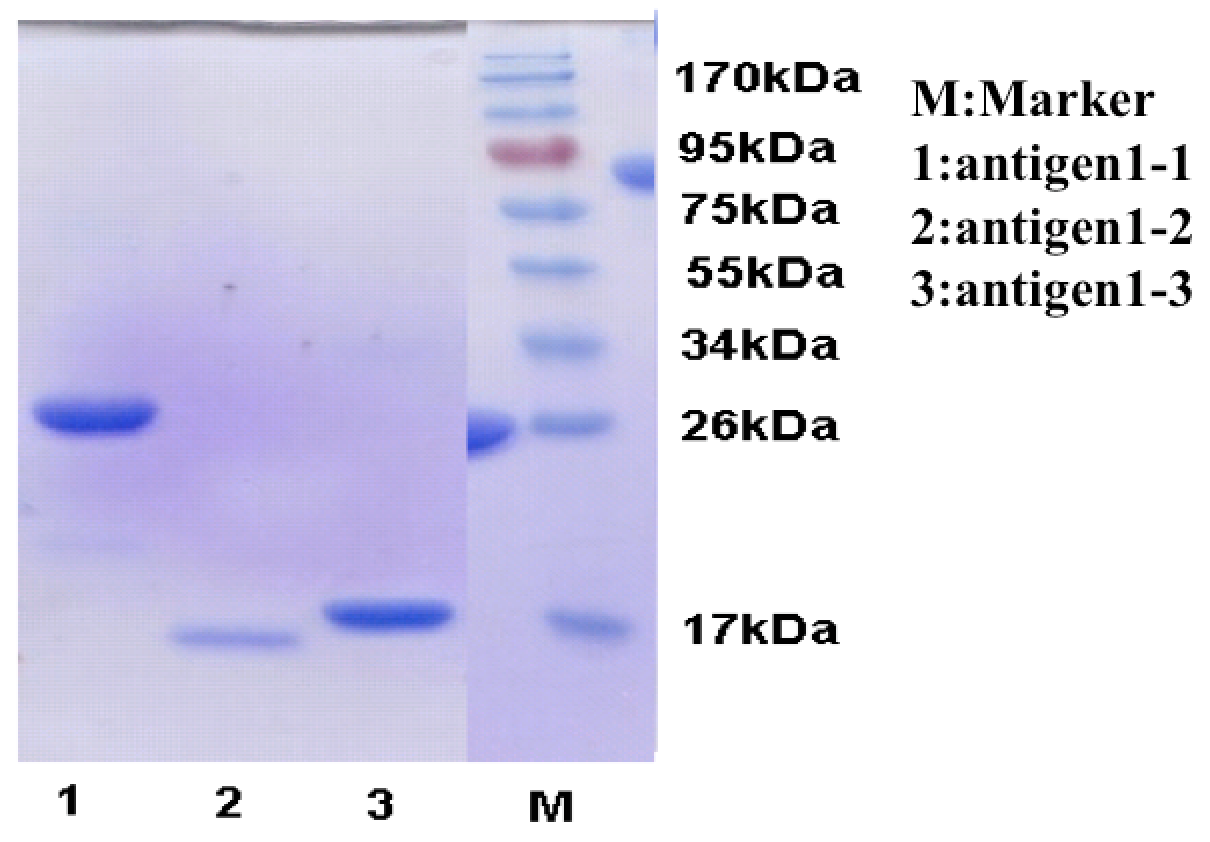


**Fig. 2 Identification of the purified antigenic proteins by SDS-PAGE**


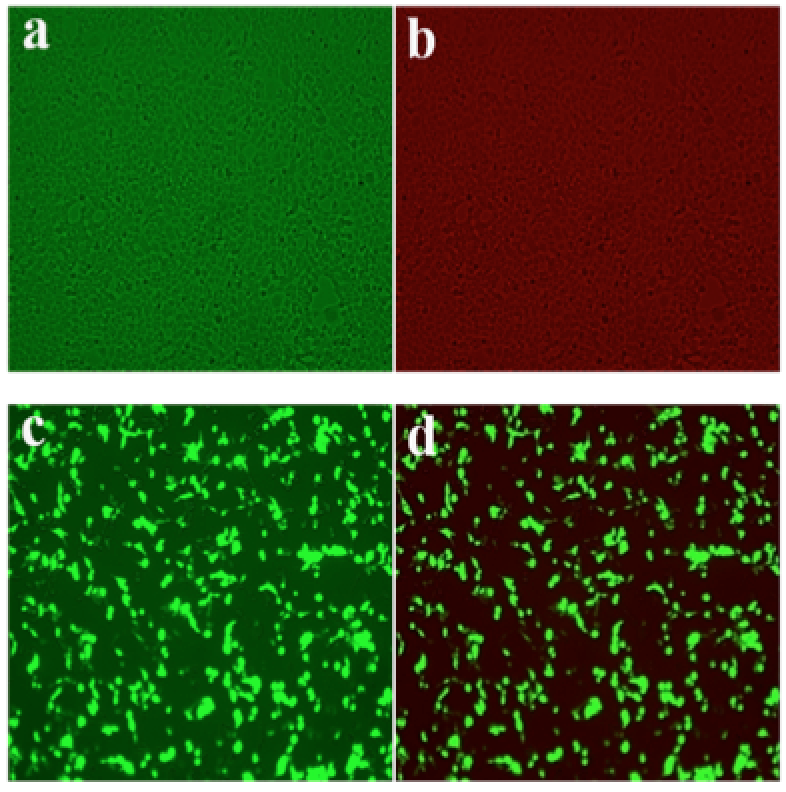


**Fig. 3 Establishment of KIT-dimer-expressing cell lines. (a) Untransfected 293 cells were observed in green background after 48 h. (b) Untransfected 293 cells were observed in red background after 48 h. (c) Transfected 293 cells were observed in green background after 48 h**. **(d) Transfected 293 cells were observed in red background after 48 h.**


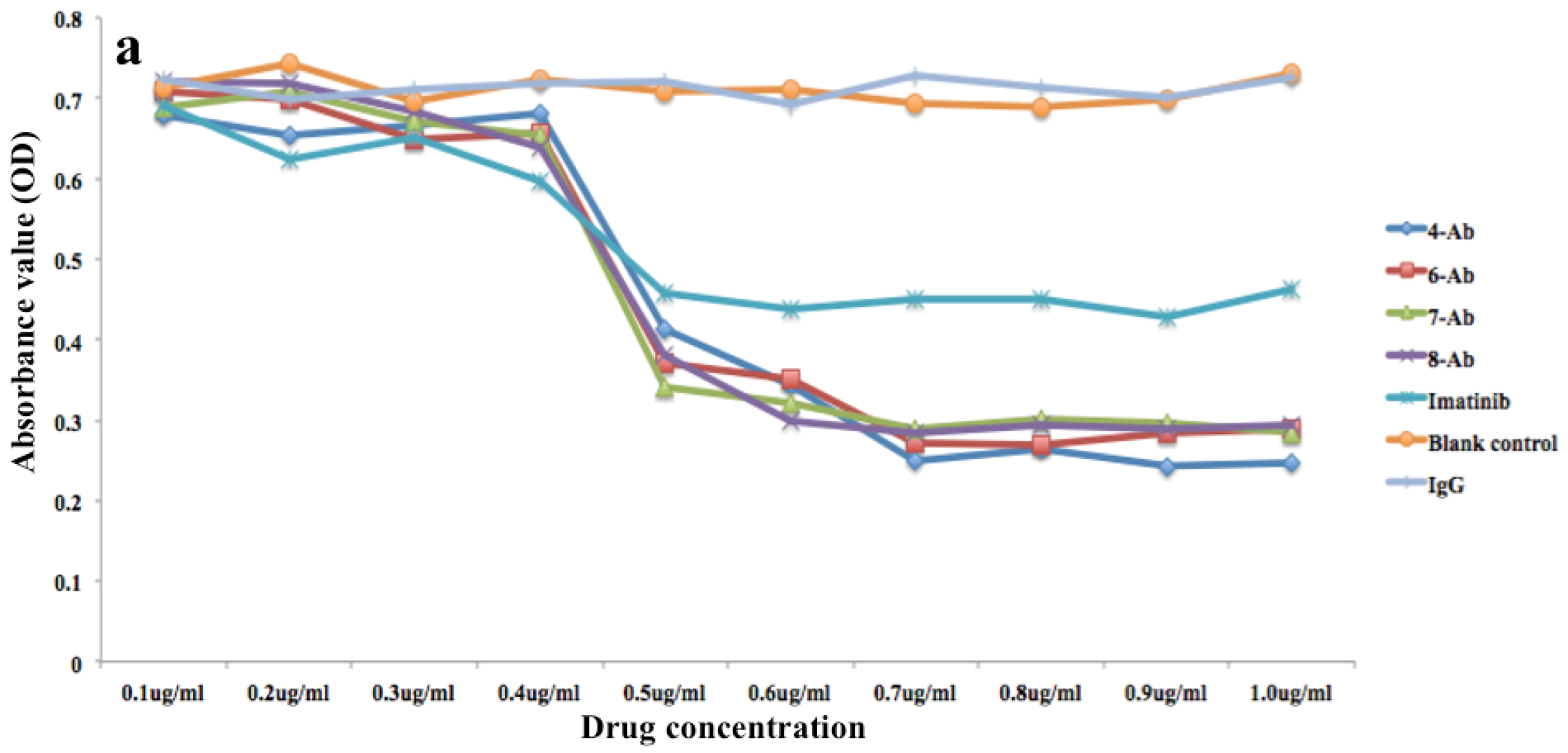


**
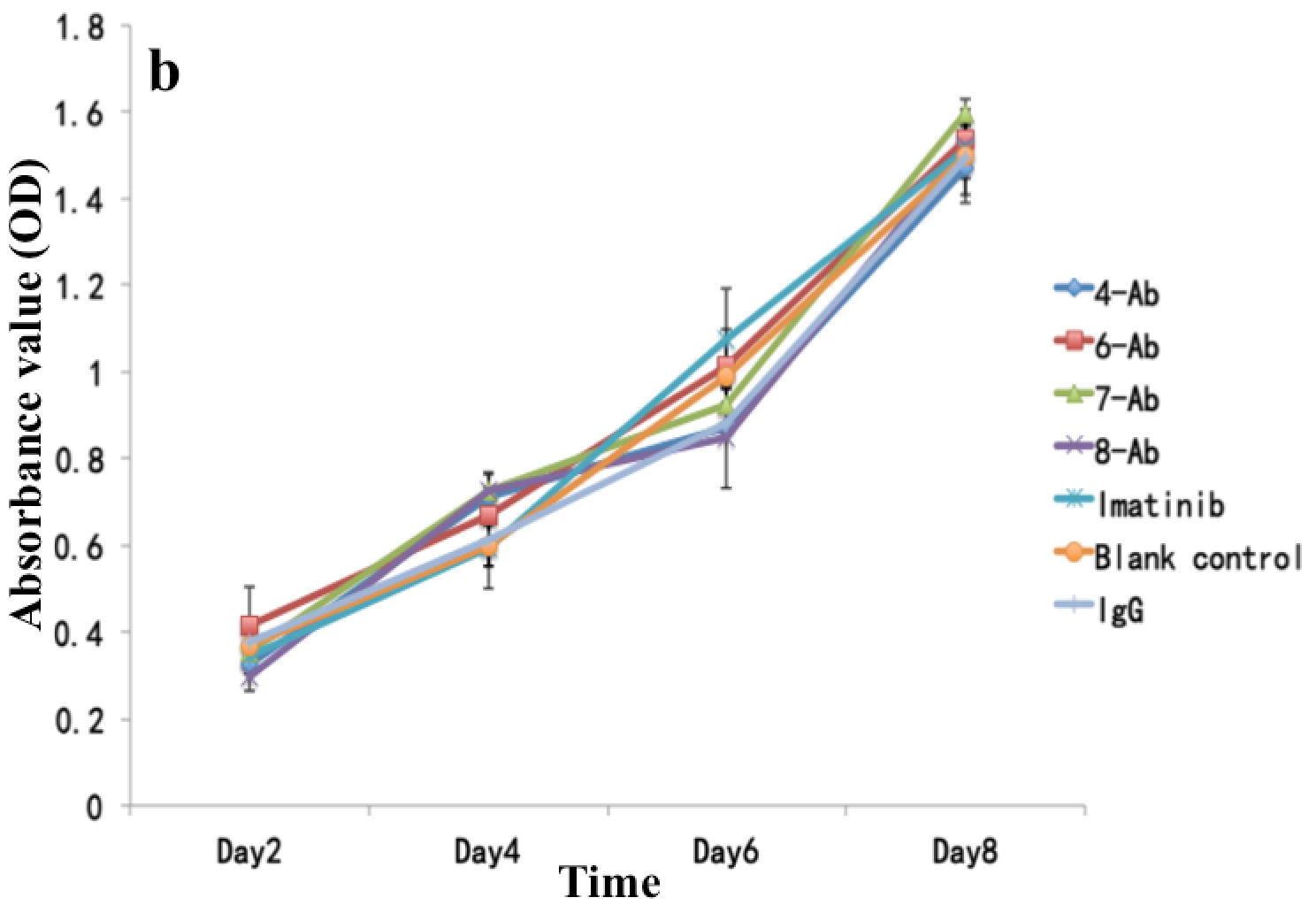
**
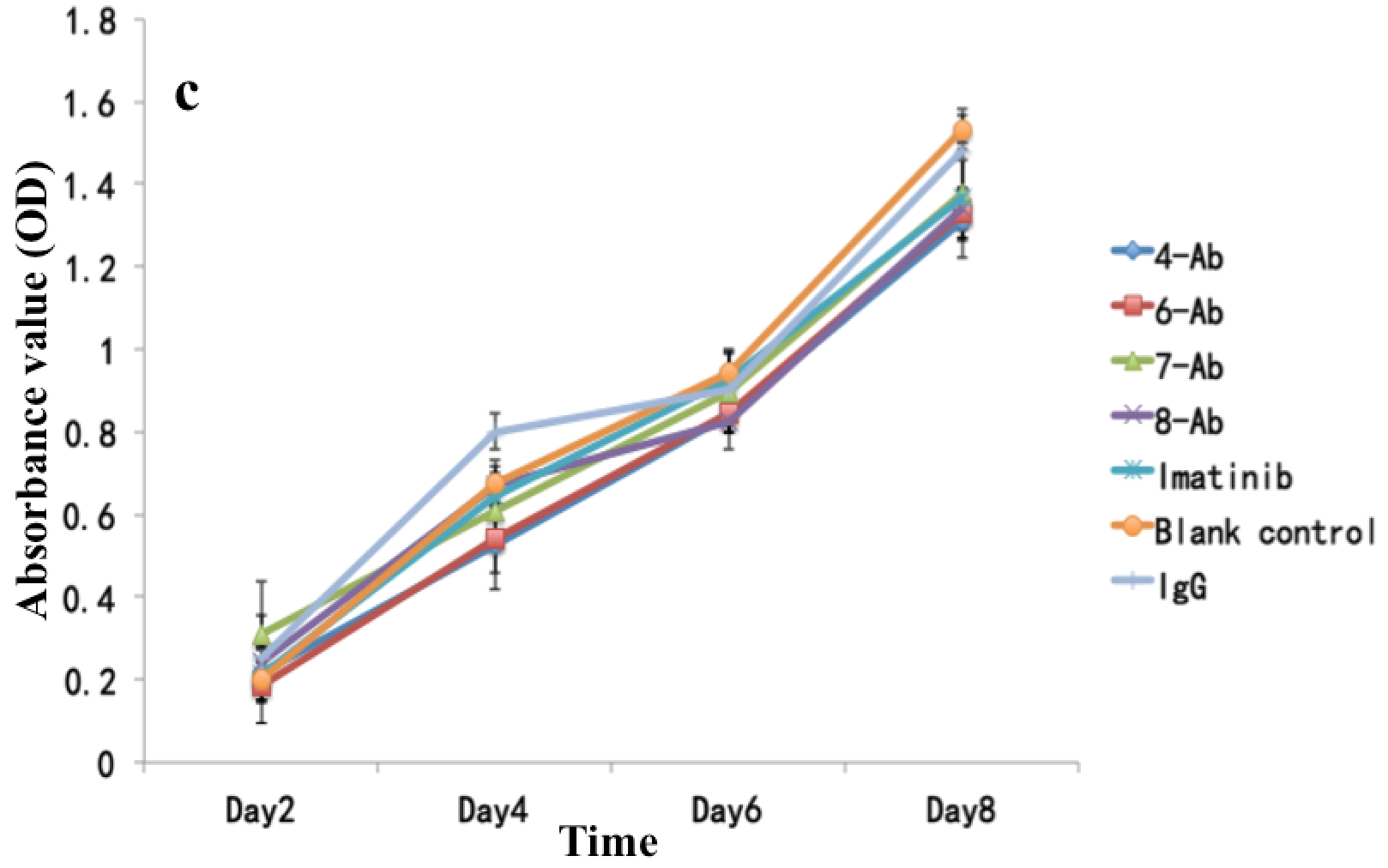


**Fig. 4 Cell proliferation of untreated, IgG-treated, imatinib-treated, and KITMAb-treated KIT-dimer-expressing cells was detected using MTT assay. (a) KIT-dimer-expressing cells were treated with gradient concentration of KITMAb for 72 h. With the concentration of KITMAb used at increasing to 0.5 μg/m, cell proliferation began to decrease significantly. (b) KITMAb and imatinib was used at the concentration of 0.1 μg/mL, respectively. There was no significant difference between the experimental groups and all the control groups. (c) KITMAb and imatinib was used at the concentration of 0.3 μg/mL, respectively. There was still no significant difference between the experimental groups and all the control groups.**
